# COVID-19-Impfstatus, genutzte Informationsquellen und soziodemografische Merkmale – Ergebnisse der CoSiD-Studie

**DOI:** 10.1007/s00103-023-03736-x

**Published:** 2023-07-12

**Authors:** Wolfgang Haß, Boris Orth, Ursula von Rüden

**Affiliations:** 1 Referat Q3 – Evaluation, Methoden, Forschungsdaten, Bundeszentrale für gesundheitliche Aufklärung (BZgA), Maarweg 149–161, 50825 Köln, Deutschland; 2 Referat G4 – Forschung und Qualitätssicherung, Bundeszentrale für gesundheitliche Aufklärung (BZgA), 50825 Köln, Deutschland

**Keywords:** COVID-19-Schutzimpfung, Impfabsicht, Soziale Unterschiede, Informationsverhalten, Repräsentativbefragung, COVID-19 vaccination, Vaccination intention, Social differences, Information, Representative survey

## Abstract

**Hintergrund:**

Trotz der Vorteile einer COVID-19-Schutzimpfung galten in Deutschland im Sommer 2022 noch immer zu wenige Menschen als geimpft. Dies wurde u. a. mit soziodemografischen Unterschieden erklärt. Der Artikel analysiert diesen Zusammenhang auch unter Einbeziehung der genutzten Informationsquellen zur Corona-Schutzimpfung anhand der Daten der dritten Erhebung der „Begleitforschung zur Kommunikation der Corona-Schutzimpfung in Deutschland“ (CoSiD-Studie, Nov./Dez. 2021; *n* = 4366 ab 16-Jährige).

**Methode:**

Es wurden bi- und multivariate Zusammenhänge zwischen der Inanspruchnahme der Impfung beziehungsweise der Impfabsicht Ungeimpfter und soziodemografischen Merkmalen sowie genutzten Informationsquellen zur COVID-19-Schutzimpfung analysiert.

**Ergebnisse:**

87,7 % der Befragten waren bereits mindestens einmal geimpft. Die Impfquote steigt tendenziell mit dem Alter, dem Bildungsgrad sowie dem Haushaltseinkommen und ist höher unter Personen in den alten Bundesländern und ohne Migrationshintergrund. Als Informationsquellen zur COVID-19-Schutzimpfung wurden zumeist Gespräche im Familien‑, Freundes- und Kollegenkreis (81,8 %) sowie deutsche TV- und Radiosender (77,1 %) genannt. Im multivariaten Modell finden sich unter den Befragten, die sich in TV- und Radiosendern aus dem Ausland und den sozialen Medien informierten, höhere Anteile von Menschen ohne Impfabsicht.

**Diskussion:**

Zielgruppenspezifische Informationsangebote müssen soziale Ungleichheiten noch stärker berücksichtigen. Hierbei ist eine Herausforderung, dass die Nutzung verschiedener Informationsquellen auch mit unterschiedlichen Impfabsichten zusammenhängt. Insbesondere Menschen mit Impfabsicht und Unentschlossene lassen sich mit gut verständlichen, vertrauenswürdigen Informationsangeboten erreichen.

## Abstract (en)

**Background:**

Despite the benefits of a COVID-19 vaccination, too few people in Germany were still considered vaccinated in the summer of 2022. This was explained, among other factors, by socio-demographic differences. The article also analyses this correlation by including the sources of information used on corona vaccination based on the data from the third survey of the “Accompanying research on the communication of corona vaccination in Germany” (CoSiD study, Nov./Dec. 2021; *n* = 4366 16-year-olds and older).

**Methods:**

Bi- and multivariate correlations were analysed between the uptake of vaccination or the intention to vaccinate of the unvaccinated and socio-demographic characteristics as well as sources of information on COVID-19 vaccination.

**Results:**

Of the respondents, 87.7% have been vaccinated at least once. The vaccination rate tends to increase with age, education level and household income and is higher among people in the old federal states and without a migration background. The sources of information on COVID-19 vaccination were mostly discussions among family, friends and colleagues (81.8%) and German TV and radio stations (77.1%). In the multivariate model, higher proportions of people without vaccination intentions were found among respondents who obtained information from TV and radio stations from abroad as well as from social media.

**Discussion:**

Information offerings for specific target groups must take social inequalities even more into account. One challenge here is that the use of different information sources is related to different vaccination intentions. In particular, people with vaccination intentions and the undecided can be reached with easily understandable, trustworthy information offerings.

## Einleitung

Die Verbreitung des COVID-19(coronavirus disease 2019)-verursachenden Virus SARS-CoV‑2 (Severe Acute Respiratory Syndrome Coronavirus 2) hat umfangreiche Verhaltensregeln wie physische Distanzierung, Tragen von Schutzmasken, Telearbeit und Quarantäne sowie insbesondere die Schutzimpfung als effektive Maßnahme zur Verminderung schwerer Krankheitsverläufe und Todesfälle nach sich gezogen [1, 2]. Hohe Impfquoten wurden aus wissenschaftlicher Sicht weitgehend übereinstimmend empfohlen. Seit Juni 2021 bestand für alle erwachsenen Personen in Deutschland die Möglichkeit, sich gegen COVID-19 impfen zu lassen. Die Impfbereitschaft wurde auf mindestens 70 % geschätzt (zzgl. 20 % Unentschlossene [3]).

Studien aus Europa und den USA deuten länderübergreifend darauf hin, dass Unterschiede in der COVID-19-Impfbereitschaft und -inanspruchnahme der Bevölkerung von mehreren Faktoren beeinflusst werden [4–10]. Es hat sich außerdem gezeigt, dass die Gruppe der Ungeimpften sehr heterogen ist und sich aus allen Teilen der Bevölkerung zusammensetzt [11, 12]. Die Gründe einer ablehnenden Haltung gegenüber dem Impfen haben sich als vielfältig und zudem im Zeitverlauf variabel erwiesen [13].

Während Frauen in internationalen Studien tendenziell eine niedrigere Impfbereitschaft aufweisen [14], haben sich in Deutschland keine nennenswerten bzw. nur geringfügige Unterschiede zwischen den Geschlechtern hinsichtlich des COVID-19-Impfstatus gezeigt [15, 16]. Für weitere soziodemografische Faktoren wie Alter, Bildung und Migrationshintergrund zeigen sich auch in Deutschland Zusammenhänge. Ältere Menschen haben ein erhöhtes Risiko für einen schweren Infektionsverlauf und eine höhere Mortalität. Dies geht einher mit einem stärkeren Bedrohungsgefühl durch COVID-19, einer positiveren Impfeinstellung und einer besseren Informiertheit über die COVID-19-Impfung. [16–19]. Ein höherer Bildungsstatus korrespondiert mit einer höheren Gesundheitskompetenz, die ebenfalls eine höhere Impfbereitschaft begünstigt [16, 20–23]. Eine hohe *allgemeine* Gesundheitskompetenz allein erscheint jedoch für eine kompetente Informationssuche unter Krisenbedingungen, z. B. im Internet, nicht hinreichend zu sein [24, 25]. Eine niedrigere Impfquote unter Menschen mit Migrationshintergrund wird in der Fachliteratur auf mangelnde Sprachkenntnisse, unzureichenden Zielgruppenzuschnitt der Informationsangebote, Diskriminierungserfahrungen im Gesundheitswesen, aber auch auf häufig geringere Bildung, geringeres Einkommen und Alter zurückgeführt [12]. In den östlichen Bundesländern zeigt sich eine tendenziell kritischere Beurteilung der Impfung von zumindest Teilen der Bevölkerung, was sich auch in entsprechend niedrigeren Impfquoten niederschlägt [26].

Psychologische, soziale und politische Determinanten spielen ebenfalls eine Rolle für unterschiedliche COVID-19-Inanspruchnahmequoten. Zu ihnen gehören Vertrauen in die Impfstoffe, Ängste hinsichtlich der unzureichenden Erforschtheit der neuen Impfstoffe sowie der Glaube an alternative Heilverfahren. Auch die Wahrnehmung des eigenen Risikos zu erkranken, das Verantwortungsgefühl für andere, Kosten-Nutzen-Abwägungen oder Erfahrungen mit anderen Impfungen (insbes. gegen Grippe) zählen dazu [14, 27–29]. Zu den sozialen Einflussfaktoren gehören Einstellungen im persönlichen Umfeld, die oft mit der eigenen Einstellung zum Impfen und zur Impfbereitschaft übereinstimmen [21, 30]. Auch politische Einstellungen, wie ein generelles Misstrauen dem Staat gegenüber, lassen sich mit einer eher skeptischen COVID-19-Impfeinstellung verknüpfen [11].

Schließlich können auch strukturelle Parameter wie die Verfügbarkeit von Impfstoffen, die räumliche Nähe zu einer Impfmöglichkeit oder andere Anreizstrukturen förderlich für die Impfbereitschaft sein [31].

Unerwartete Ereignisse wie der Ausbruch von Pandemien erfordern ein hohes Maß an individueller Anpassung, um diese zu bewältigen, und führen zu einem gesteigerten Informationsbedarf. Entsprechend hat die Informationssuche während der COVID-19-Pandemie zugenommen, wobei vielerlei Quellen genutzt wurden [32]. Während der Abruf zuverlässiger und qualitativ hochwertiger Informationen eher von öffentlichen Institutionen, aus dem Radio und Fernsehen oder aus nationalen und lokalen Zeitungen erfolgte, wurden interpersonale Kontakte und soziale Medien eher für Austausch, Meinungsbildung und emotionale Unterstützung genutzt [21, 33, 34]. Studien zeigen, dass Personen, die sich stärker bedroht fühlen, Medien zwar häufiger, aber mit einer geringeren Bandbreite nutzen und sich besser informiert fühlen. Dies korrespondiert allerdings nicht unbedingt mit ihrem tatsächlichen Wissen; möglicherweise wird der eigene Wissensstand überschätzt [35, 36]. Generell scheint eine bessere Informiertheit mit einer höheren Akzeptanz von Maßnahmen (bspw. Quarantäne) sowie regelkonformem Verhalten einherzugehen [37].

Dem hohen Informationsbedarf entsprechend gab es einen exponentiellen Anstieg an Informationen zu COVID-19 und der Impfung in unterschiedlichen Medien. Diese wiesen jedoch nicht immer die üblichen Qualitätsstandards wissenschaftlicher Informationen auf. So kursierten häufig vorläufige, ungeprüfte, uneinheitliche, zum Teil auch widersprüchliche oder gar falsche Informationen [38], was aufseiten der Adressaten zu Unsicherheiten beitrug. Insbesondere im Internet, in dem Postings in sozialen Medien sowie Nachrichten, Meinungen und vieles Weitere oft ungeordnet nebeneinanderstehen, ist es oft schwierig, richtige von falschen oder irreführenden Informationen zu unterscheiden. So ließen sich in COVID-19-bezogenen Postings Anteile von bis zu 41 % als Fehlinformationen identifizieren [39, 40].

Solche Fehlinformationen tragen zu Zweifeln an der Sicherheit von Impfstoffen und an offiziellen Schutzregeln bei sowie zu einer geringeren Impfabsicht [41–43]. Beispielsweise nahmen bei einer Befragung türkischsprachiger Menschen in Deutschland knapp 12 % fälschlicherweise an, dass sie sich eine COVID-19-Impfung finanziell nicht leisten können, obwohl diese kostenfrei war [44]. Die Weltgesundheitsorganisation (WHO; [45]) warnt davor, dass eine „Infodemie“ als Kombination aus Informationsüberangebot und zweifelhaften Informationsquellen zu Verwirrungen und riskanten gesundheitsschädlichen Verhaltensweisen führen kann (zum Beispiel bezüglich psychischer Gesundheit: [46]) und das Misstrauen gegenüber den Einrichtungen des Gesundheitswesens verstärken kann. Eine Infodemie kann zu Überforderung und damit auch zu Informationsvermeidungsverhalten führen [47].

Vor diesem Hintergrund geht der vorliegende Beitrag der Frage nach, welche Zusammenhänge sich zwischen dem Impfstatus bzw. der Impfabsicht und soziodemografischen Faktoren einerseits sowie darüber hinaus der Nutzung verschiedener Informationsquellen aufzeigen lassen. Zugrunde gelegt werden dafür Daten der CoSiD-Studie, die repräsentative Ergebnisse zu Wissen, Einstellungen, Informiertheit und Verhalten der Bevölkerung in Bezug auf die COVID-19-Schutzimpfung lieferte [20].

## Methoden

### Studie, Stichprobenziehung und Datenerhebung

Dieser Beitrag beruht auf den Daten der dritten Befragung der Begleitforschung zur Kommunikation der Corona-Schutzimpfung in Deutschland (CoSiD), die die Bundeszentrale für gesundheitliche Aufklärung (BZgA) im Jahr 2021 durchgeführt hat. Die CoSiD-Studie ist eine deutschlandweite, wiederholte Repräsentativbefragung zu Wissen, Einstellungen sowie Informations- und Impfverhalten der Allgemeinbevölkerung bezogen auf die Corona-Schutzimpfung. Die dritte Befragung erfolgte vom 15.11. bis zum 08.12.2021.

Grundgesamtheit der Befragung war die deutschsprachige Wohnbevölkerung ab 16 Jahren, die in Privathaushalten lebte. Stichprobenziehung und Datenerhebung erfolgten im Mixed-Mode-Ansatz. Unabhängig voneinander wurden eine Telefon- und eine Online-Stichprobe zufällig gezogen und telefonisch (CATI = Computer-assisted Telephone Interviewing) beziehungsweise online (CAWI = Computer-assisted Web Interviewing) befragt. Die Telefonstichprobe war eine Dual-Frame-Stichprobe, in der eine Teilstichprobe auf Basis von Festnetztelefonnummern und eine Teilstichprobe auf Basis von Mobiltelefonnummern miteinander kombiniert wurden. Die Stichprobe der Online-Befragung wurde zufällig aus einem aktiven Online-Access-Panel gezogen. Über das Online-Panel wurde eine disproportionale Aufstockung von ungeimpften Menschen und von Menschen mit Migrationshintergrund realisiert. Stichprobenziehung und Datenerhebung wurden von der INFO GmbH Markt- und Meinungsforschung, Berlin, im Auftrag der BZgA durchgeführt. Alle Befragten wurden vor Start des Interviews über die Ziele der Studie, die Freiwilligkeit der Teilnahme sowie die Anonymisierung und den Datenschutz aufgeklärt. Die telefonischen Interviews dauerten durchschnittlich 30 min; die Online-Befragungen dauerten durchschnittlich 24 min.

### Untersuchte Variablen

#### Impfstatus und -absicht

In der CoSiD-Studie wurden sowohl der Impfstatus („Haben Sie bereits Ihre Corona-Schutzimpfung erhalten?“) als auch die Impfabsicht ungeimpfter Menschen („Haben Sie vor, sich gegen das Coronavirus impfen zu lassen?“) erfragt. Damit wurde sowohl die Ebene individuellen Verhaltens als auch die der Verhaltensintention angesprochen. Aus beiden Variablen wurde ein Indikator gebildet, der geimpfte von ungeimpften Menschen unterscheidet und ungeimpfte Menschen auf Ebene ihrer Verhaltensintention weiter ausdifferenziert. Das ergab eine abhängige Variable mit 4 Kategorien: (1) Menschen mit mindestens einer Impfung, (2) ungeimpfte Menschen, die sich „auf jeden Fall“ beziehungsweise „eher“ impfen lassen wollen, (3) ungeimpfte Menschen, die in der Frage des Impfens „unentschlossen“ sind, und (4) ungeimpfte Menschen, die sich „eher nicht“ beziehungsweise „auf keinen Fall“ impfen lassen wollen.

#### Soziodemografische Indikatoren

Als soziodemografische Variablen wurden in die Analysen aufgenommen: Alter (in Jahren), Geschlecht (männlich, weiblich, divers), Bildungsstand (maximal Hauptschulabschluss, Realschulabschluss, (Fach‑)Hochschulreife, (Fach‑)Hochschulabschluss), Haushaltsnettoeinkommen (keine Angabe, weniger als 60 %, 60 % bis weniger als 150 % sowie 150 % und mehr des Medians des Nettoäquivalenzeinkommens in Deutschland), Migrationshintergrund (Befragte oder mindestens ein Elternteil haben/hatten eine andere als die deutsche Staatsbürgerschaft) sowie die Region (ost- und westdeutsche Bundesländer).

#### Nutzung verschiedener Informationsmöglichkeiten

Die Nutzung verschiedener Informationsmöglichkeiten wurde mit der Frage: „Hier kommen nun einige Möglichkeiten, sich über die Corona-Schutzimpfung zu informieren. Bitte geben Sie an, wie häufig Sie die jeweiligen Informationsquellen in den letzten 30 Tagen genutzt haben“, ermittelt. Es wurden 10 potenzielle Informationsquellen vorgegeben (zum Beispiel eher informelle Gespräche im Familien‑, Freundes- oder Kollegenkreis, Informationsangebote der klassischen und der sozialen Medien oder von staatlichen Einrichtungen). Die Antworten erfolgten auf einer 5‑stufigen Likert-Skala von „sehr häufig“ über „häufig“, „gelegentlich“, „selten“ bis „nie“. Die Befragten wurden in 2 Gruppen eingeteilt: Befragte, die die jeweilige Informationsquelle zumindest gelegentlich verwendet hatten, sowie Befragte, die die jeweilige Informationsquelle selten beziehungsweise nie genutzt hatten.

### Statistische Auswertung

In der vorliegenden Untersuchung wurden die Daten repräsentativ für die Allgemeinbevölkerung gewichtet und ausgewertet. Für die Gewichtung der Telefonstichprobe wurde eine Dual-Frame-Designgewichtung berechnet, die die unterschiedliche Auswahlwahrscheinlichkeit der Befragten aufgrund unterschiedlich vieler Festnetz- und Mobilfunknummern sowie Personen im Haushalt ausgleicht. Zudem wurde die Gesamtstichprobe nach den Merkmalen Alter, Geschlecht, Schulbildung, Haushaltsgröße, Bundesland sowie Migrationshintergrund gewichtet. Schließlich wurden die disproportionalen Aufstockungen der Menschen mit Migrationshintergrund und der ungeimpften Menschen auf die Anteile in der Stichprobe ohne Aufstockung heruntergewichtet.

Die Auswertung erfolgte mit dem Modul „Complex Samples“ der Analysesoftware IBM SPSS Statistics 26.0 (IBM, New York, USA). Die uni- und bivariaten Häufigkeitsverteilungen kategorialer Variablen wurden in Prozentwerten abgebildet und die dazugehörigen 2‑seitigen 95 %-Konfidenzintervalle bestimmt. Multivariate Zusammenhangsanalysen wurden mit einem multinomialen logistischen Regressionsmodell berechnet. Abhängige Variable ist der beschriebene Indikator zum Impfstatus bzw. zu den Impfabsichten. Als Referenzgruppe diente die Gruppe der geimpften Menschen und es wurde getestet, ob sich im Verhältnis zu dieser Gruppe die Anteile ungeimpfter Menschen, die entweder für die Impfung, unentschlossen oder gegen die Impfung waren, in Abhängigkeit von unterschiedlichen Ausprägungen der Prädiktorvariablen unterscheiden. Prädiktorvariablen im Modell sind die soziodemografischen sowie die dichotomen Variablen der verschiedenen Informationsmöglichkeiten zur Corona-Schutzimpfung. Es werden die Regressionskoeffizienten inklusive Konfidenzintervalle und *p*-Werte berichtet. Das Signifikanzniveau wurde auf *p* < 0,05 festgesetzt.

## Ergebnisse

### Stichprobenbeschreibung

An der Befragung beteiligten sich 4366 Menschen im Alter von 16–93 Jahren. 728 nahmen über Festnetztelefon (16,7 %), 788 über Mobiltelefon (18,0 %) und 2850 online (65,3 %) teil. Die Online-Stichprobe umfasste auch die Aufstockungen um 792 ungeimpfte Menschen und 1516 Menschen mit Migrationshintergrund. 10 Befragte gaben als Geschlecht „divers“ an. Da diese Gruppe zu klein war, um sie in die multiple Regressionsanalyse (s. unten) einbeziehen zu können, wurde sie in den Auswertungen dieses Beitrags nicht berücksichtigt, so dass die hier verwendete Analysestichprobe ungewichtet 4356 Befragte umfasst (Tab. 1).

**Table 1:** 

		Absolute Häufigkeit (ungewichtetes *n*)	Absolute Häufigkeit (gewichtetes *n*)	Relative Häufigkeit (gewichtete %)
Insgesamt		4356	4359	100,0
Alter	16 bis 39 Jahre	1744	1453	33,3
	40 bis 65 Jahre	1917	1860	42,7
	66 Jahre und älter	695	1047	24,0
Geschlecht	Männlich	2111	2133	48,9
	Weiblich	2245	2227	51,1
Bildung	Max. Hauptschulabschluss/WN/KA	648	836	19,2
	Realschulabschluss	1646	1926	44,2
	(Fach‑)Hochschulreife	906	719	16,5
	(Fach‑)Hochschulabschluss	1156	878	20,1
Migrationshintergrund	Nein	2362	3812	87,5
	Ja	1994	547	12,5
Nettoäquivalenzeinkommen	Keine Angabe	357	445	10,2
	< 60 % Median	1150	938	21,5
	60 % bis < 150 % Median	2326	2414	55,4
	≥ 150 % Median	523	563	12,9
Region	Alte Bundesländer^a^	3597	3593	82,4
	Neue Bundesländer^b^	759	766	17,6

*WN* weiß nicht, *KA* keine Angabe

^a^Einschließlich Westberlin

^b^Einschließlich Ostberlin

Durch die Gewichtung der Daten, die die Stichprobenverteilung an die statistische Verteilung in der Bevölkerung anpasst, wurde die Anzahl jüngerer Menschen verringert und die Anzahl älterer Menschen erhöht. Die Bildungsverteilung veränderte sich zugunsten der Menschen mit Haupt- oder Realschulabschluss. Außerdem korrigiert die Gewichtung die im Stichprobendesign angelegte überproportionale Berücksichtigung von Menschen mit Migrationshintergrund (Tab. 1) und ungeimpften Menschen (Tab. 2).

**Table 2:** 

		Geimpft		Ungeimpft und auf jeden Fall/eher impfen		Ungeimpft und unentschlossen		Ungeimpft und eher nicht/auf keinen Fall impfen	
	Ungewichtete Fallzahlen	*n* = 3064		*n* = 230		*n* = 286		*n* = 776	
	Gewichtete Fallzahlen	*n* = 3825		*n* = 88		*n* = 114		*n* = 332	
		%	(95 %-KI)	%	(95 %-KI)	%	(95 %-KI)	%	(95 %-KI)
Insgesamt		87,7	(87,3; 88,2)	2,0	(1,7; 2,4)	2,6	(2,3; 3,0)	7,6	(7,2; 8,1)
Alter	16 bis 39 Jahre	81,4	(79,7; 83,1)	3,4	(2,8; 4,2)	4,6	(3,8; 5,5)	10,6	(9,4; 12,0)
	40 bis 65 Jahre	88,4	(87,4; 89,3)	1,6	(1,2; 2,0)	2,2	(1,8; 2,7)	7,8	(7,0; 8,7)
	66 Jahre und älter	95,3	(93,7; 96,5)	0,9	(0,4; 1,9)	0,6	(0,3; 1,3)	3,2	(2,2; 4,4)
Geschlecht	Männlich	87,5	(86,4; 88,5)	2,0	(1,6; 2,5)	2,5	(2,0; 3,0)	8,0	(7,2; 9,0)
	Weiblich	88,0	(87,0; 88,9)	2,0	(1,6; 2,6)	2,8	(2,3; 3,3)	7,2	(6,5; 8,0)
Bildung	Max. Hauptschulabschluss/WN/KA	87,3	(85,2; 89,2)	2,0	(1,4; 2,8)	2,9	(2,1; 4,0)	7,8	(6,4; 9,4)
	Realschulabschluss	84,8	(83,5; 86,0)	2,3	(1,9; 2,9)	3,0	(2,5; 3,7)	9,8	(8,8; 10,9)
	(Fach‑)Hochschulreife	87,7	(85,4; 89,6)	2,7	(1,8; 4,0)	2,9	(2,1; 4,0)	6,7	(5,4; 8,4)
	(Fach‑)Hochschulabschluss	94,5	(93,1; 95,6)	0,9	(0,6; 1,4)	1,2	(0,8; 1,9)	3,4	(2,5; 4,6)
Migrationshintergrund	Nein	88,6	(88,2; 89,1)	1,7	(1,4; 2,0)	2,3	(1,9; 2,6)	7,4	(7,0; 8,0)
	Ja	81,4	(80,4; 82,3)	4,6	(3,8; 5,6)	5,2	(4,4; 6,2)	8,8	(7,8; 10,0)
Nettoäquivalenzeinkommen	Keine Angabe	91,4	(88,6; 93,6)	2,3	(1,2; 4,4)	1,2	(0,6; 2,2)	5,1	(3,6; 7,2)
	< 60 % Median	81,7	(79,5; 83,7)	3,2	(2,5; 4,1)	4,1	(3,3; 5,1)	11,0	(9,5; 12,7)
	60 % bis < 150 % Median	88,0	(87,1; 88,9)	1,9	(1,6; 2,4)	2,6	(2,2; 3,1)	7,5	(6,7; 8,3)
	≥ 150 % Median	93,6	(91,7; 95,1)	0,2	(0,1; 0,7)	1,4	(0,8; 2,5)	4,7	(3,5; 6,4)
Region	Alte Bundesländer^a^	88,6	(88,0; 89,2)	2,1	(1,8; 2,5)	2,6	(2,2; 3,0)	6,7	(6,2; 7,3)
	Neue Bundesländer^b^	83,6	(81,3; 85,6)	1,7	(1,2; 2,4)	2,9	(2,1; 4,0)	11,8	(10,1; 13,7)

*% (95* *%-KI)* gewichtete Prozentangaben mit 95 %-Konfidenzintervall, *WN* weiß nicht, *KA* keine Angabe

^a^Einschließlich Westberlin

^b^Einschließlich Ostberlin

In der gewichteten Stichprobe verfügten 19,2 % der Befragten maximal über einen Hauptschulabschluss, 12,5 % hatten einen Migrationshintergrund und 21,5 % lebten in Haushalten mit weniger als 60 % des Nettoäquivalenzeinkommens in Deutschland, das heißt unter der Armutsgrenze (Tab. 1).

Im Folgenden wird zunächst die Nutzung verschiedener Informationsmöglichkeiten zur COVID-19-Schutzimpfung dargestellt. Danach wird der Impfstatus differenziert nach soziodemografischen Variablen und nach dem Informationsverhalten aufgezeigt. Abschließend wird der Frage nachgegangen, wie sich der jeweils erhobene Impfstatus erklären lässt, wenn nicht nur soziodemografische Variablen, sondern auch die genutzten Informationsquellen zur COVID-19-Schutzimpfung herangezogen werden. Dazu wird ein multiples Regressionsmodell präsentiert, das die simultane Einbeziehung mehrerer Variablen ermöglicht.

### Nutzung verschiedener Informationsmöglichkeiten zur COVID-19-Schutzimpfung

**Figure 1:**
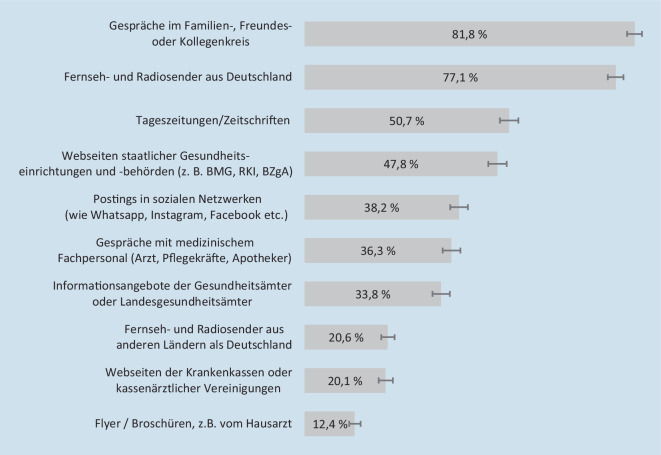

In den letzten 30 Tagen vor der Befragung hatten etwa 4 Fünftel aller Befragten zumindest gelegentlich im Freundes‑, Familien- oder Kollegenkreis Gespräche über die COVID-19-Schutzimpfung geführt (81,8 % (95 %-KI: 79,8–83,6 %)) oder sich über Fernseh- und Radiosender aus Deutschland informiert (77,1 % (95 %-KI: 75,1–79,0 %); Abb. 1). Etwa die Hälfte hatte sich zumindest gelegentlich in Tageszeitungen und Zeitschriften (50,7 % (95 %-KI: 48,4–53,0 %)) oder auf Webseiten staatlicher Gesundheitseinrichtungen und -behörden wie dem Bundesministerium für Gesundheit (BMG), dem Robert Koch-Institut (RKI) oder der BZgA informiert (47,8 % (95 %-KI: 45,5–50,1 %)). Knapp 2 Fünftel hatten Postings in sozialen Netzwerken genutzt (38,2 % (95 %-KI: 36,1–40,5 %)) oder Gespräche mit medizinischem Fachpersonal geführt (36,3 % (95 %-KI: 34,2–38,6 %)), um sich über die COVID-19-Schutzimpfung zu informieren. Flyer oder Broschüren, zum Beispiel vom Hausarzt, hatte lediglich etwa jeder Achte genutzt (12,4 % (95 %-KI: 11,0–13,9 %)).

### COVID-19-Impfinanspruchnahme bzw. -absicht und soziodemografische Merkmale

Es kann davon ausgegangen werden, dass jede/jeder Impfbereite in Deutschland zum Zeitpunkt der Befragung auch die Möglichkeit gehabt hatte, sich gegen COVID-19 impfen zu lassen. Insgesamt waren 87,7 % der Befragten mindestens einmal geimpft; 2,0 % waren ungeimpft und wollten sich noch impfen lassen; 2,6 % waren ungeimpft und unentschlossen. 7,6 %, also die Mehrheit der Ungeimpften, wollten sich nicht impfen lassen (Tab. 2). Der Anteil geimpfter Menschen stieg mit dem Alter und war bei Menschen mit (Fach‑)Hochschulabschluss höher als bei Menschen ohne (Fach‑)Hochschulabschluss. Von den Menschen ohne Migrationshintergrund waren mehr geimpft als von den Menschen mit Migrationshintergrund. Des Weiteren hatten Menschen mit höherem Haushaltseinkommen die Möglichkeit, sich impfen zu lassen, mehr in Anspruch genommen und in den alten Bundesländern waren mehr Menschen geimpft als in den neuen Bundesländern.

### COVID-19-Impfinanspruchnahme bzw. -absicht und die Nutzung verschiedener Informationsmöglichkeiten

Der Anteil geimpfter Menschen war höher, wenn diese sich in den letzten 30 Tagen zumindest gelegentlich über TV- und Radiosender aus Deutschland, in Tageszeitungen und Zeitschriften und in Gesprächen mit medizinischem Fachpersonal über die COVID-19-Schutzimpfung informiert hatten (Tab. 3). Er war dagegen geringer, wenn sie in den letzten 30 Tagen zumindest gelegentlich Postings in sozialen Netzwerken oder TV- und Radiosender aus anderen Ländern als Deutschland genutzt hatten, um sich über die Impfung zu informieren.

**Table 3:** 

		Geimpft		Ungeimpft und auf jeden Fall/eher impfen		Ungeimpft und unentschlossen		Ungeimpft und eher nicht/auf keinen Fall impfen	
	Ungewichtete Fallzahlen	*n* = 3064		*n* = 230		*n* = 286		*n* = 776	
	Gewichtete Fallzahlen	*n* = 3825		*n* = 88		*n* = 114		*n* = 332	
		%	(95 %-KI)	%	(95 %-KI)	%	(95 %-KI)	%	(95 %-KI)
Insgesamt		87,7	(87,3; 88,2)	2,0	(1,7; 2,4)	2,6	(2,3; 3,0)	7,6	(7,2; 8,1)
Gespräche mit Familie/Freunden/Kollegen	Mindestens gelegentlich	88,1	(87,4; 88,7)	2,2	(1,8; 2,6)	2,5	(2,2; 2,9)	7,3	(6,7; 7,8)
	Nie/selten	86,2	(84,0; 88,2)	1,4	(0,9; 2,1)	3,2	(2,4; 4,3)	9,2	(7,7; 11,1)
Fernsehen/Radio aus Deutschland	Mindestens gelegentlich	89,9	(89,3; 90,6)	2,1	(1,8; 2,6)	2,4	(2,0; 2,8)	5,5	(5,0; 6,1)
	Nie/selten	80,3	(78,0; 82,4)	1,6	(1,2; 2,3)	3,4	(2,7; 4,3)	14,7	(12,9; 16,6)
Zeitungen/Zeitschriften	Mindestens gelegentlich	92,0	(91,1; 92,8)	1,7	(1,3; 2,2)	1,9	(1,5; 2,4)	4,4	(3,8; 5,1)
	Nie/selten	83,3	(82,2; 84,5)	2,4	(2,0; 2,9)	3,4	(2,8; 4,0)	10,9	(9,9; 11,9)
Staatliche Webseiten	Mindestens gelegentlich	88,8	(87,8; 89,7)	2,0	(1,6; 2,5)	2,7	(2,2; 3,3)	6,5	(5,8; 7,3)
	Nie/selten	86,8	(85,7; 87,8)	2,1	(1,7; 2,6)	2,5	(2,1; 3,0)	8,6	(7,8; 9,5)
Soziale Medien	Mindestens gelegentlich	82,7	(81,2; 84,0)	2,9	(2,3; 3,5)	3,6	(3,0; 4,3)	10,9	(9,9; 12,1)
	Nie/selten	90,9	(90,1; 91,6)	1,5	(1,2; 1,9)	2,0	(1,7; 2,5)	5,6	(5,0; 6,3)
Gespräche mit medizinischem Fachpersonal	Mindestens gelegentlich	87,7	(86,3; 88,9)	2,2	(1,7; 3,0)	2,6	(2,0; 3,2)	7,6	(6,6; 8,6)
	Nie/selten	87,8	(87,0; 88,5)	1,9	(1,6; 2,3)	2,7	(2,3; 3,1)	7,7	(7,0; 8,4)
Informationen der Gesundheitsämter	Mindestens gelegentlich	88,3	(87,0; 89,5)	2,5	(1,9; 3,1)	3,0	(2,4; 3,7)	6,3	(5,4; 7,2)
	Nie/selten	87,4	(86,6; 88,2)	1,8	(1,5; 2,2)	2,5	(2,1; 2,9)	8,3	(7,6; 9,1)
Fernsehen/Radio aus Ausland	Mindestens gelegentlich	80,0	(77,7; 82,1)	3,6	(2,7; 4,6)	3,5	(2,7; 4,4)	13,0	(11,3; 14,9)
	Nie/selten	89,7	(89,1; 90,3)	1,6	(1,3; 2,0)	2,4	(2,1; 2,8)	6,2	(5,7; 6,8)
Webseiten von Krankenkassen	Mindestens gelegentlich	86,4	(84,4; 88,2)	2,8	(2,1; 3,8)	3,3	(2,4; 4,4)	7,5	(6,3; 9,0)
	Nie/selten	88,1	(87,4; 88,7)	1,8	(1,5; 2,2)	2,5	(2,1; 2,9)	7,6	(7,1; 8,3)
Flyer/Broschüren, z. B. vom Arzt	Mindestens gelegentlich	86,4	(83,8; 88,7)	3,1	(2,1; 4,5)	3,3	(2,3; 4,6)	7,2	(5,7; 9,1)
	Nie/selten	87,9	(87,4; 88,5)	1,9	(1,6; 2,2)	2,5	(2,2; 2,9)	7,7	(7,2; 8,2)

*% (95* *%-KI)* gewichtete Prozentangaben mit 95 %-Konfidenzintervall

### Multivariate Zusammenhänge

Mit einem multiplen multinomialen Regressionsmodell wurde untersucht, ob neben soziodemografischen Merkmalen auch die Nutzung verschiedener Informationsmöglichkeiten einen Beitrag zur Erklärung des Impfstatus und der Impfabsichten leisten kann (Tab. 4).

**Table 4:** 

	Impfabsicht					
Unabhängige Variablen	Auf jeden Fall/eher impfen		Unentschlossen		Eher nicht/auf keinen Fall	
	β (95 %-KI)	*p*	β (95 %-KI)	*p*	β (95 %-KI)	*p*
Konstante	−5,34 (−6,92; −3,76)	0,000	−2,22 (−3,26; −1,18)	0,000	−2,21 (−2,96; −1,47)	0,000
Alter	−0,03 (−0,04; −0,01)	0,001	−0,03 (−0,04; −0,02)	0,000	−0,01 (−0,02; 0,00)	0,009
Männlich^a^	0,03 (−0,33; 0,40)	0,858	−0,15 (−0,46; 0,16)	0,337	0,11 (−0,12; 0,33)	0,367
Max. Hauptschulabschluss, weiß nicht, keine Angabe^b^	0,71 (0,12; 1,31)	0,019	1,05 (0,44; 1,66)	0,001	0,91 (0,44; 1,37)	0,000
Realschulabschluss^b^	0,83 (0,29; 1,36)	0,003	0,87 (0,33; 1,41)	0,002	1,02 (0,62; 1,42)	0,000
(Fach‑)Hochschulreife^b^	0,62 (−0,11; 1,34)	0,095	0,45 (−0,18; 1,08)	0,166	0,44 (−0,03; 0,91)	0,067
Ohne Migrationshintergrund^c^	−0,65 (−0,98; −0,31)	0,000	−0,56 (−0,85; −0,27)	0,000	0,16 (−0,06; 0,37)	0,148
Keine Angabe zum Einkommen^d^	2,23 (0,95; 3,51)	0,001	−0,30 (−1,22; 0,61)	0,516	−0,01 (−0,55; 0,54)	0,981
< 60 % Median/Nettoäquivalenzeinkommen^d^	2,37 (1,23; 3,51)	0,000	0,69 (0,04; 1,34)	0,038	0,38 (−0,05; 0,80)	0,084
60 % bis < 150 % Median/Nettoäquivalenzeinkommen^d^	1,97 (0,86; 3,08)	0,000	0,43 (−0,19; 1,06)	0,176	0,17 (−0,21; 0,56)	0,381
Alte Bundesländer, einschl. Westberlin ^e^	0,06 (−0,38; 0,50)	0,791	−0,23 (−0,63; 0,17)	0,257	−0,61 (−0,88; −0,33)	0,000
Mindestens gelegentlich Gespräche mit Familie/Freunden/Kollegen^f^	0,33 (−0,25; 0,92)	0,264	−0,28 (−0,73; 0,16)	0,213	0,04 (−0,28; 0,36)	0,814
Mindestens gelegentlich Nutzung von Fernsehen/Radio aus Deutschland^f^	0,34 (−0,11; 0,79)	0,142	−0,09 (−0,48; 0,29)	0,631	−1,06 (−1,34; −0,79)	0,000
Mindestens gelegentlich Nutzung von Zeitungen/Zeitschriften^f^	−0,31 (−0,72; 0,09)	0,125	−0,33 (−0,69; 0,04)	0,077	−0,60 (−0,88; −0,32)	0,000
Mindestens gelegentlich Nutzung von staatlichen Webseiten^f^	−0,54 (−0,97; −0,11)	0,014	−0,09 (−0,43; 0,25)	0,601	−0,19 (−0,45; 0,08)	0,175
Mindestens gelegentlich Nutzung von sozialen Medien^f^	0,23 (−0,17; 0,63)	0,254	0,23 (−0,13; 0,58)	0,216	0,74 (0,49; 1,00)	0,000
Mindestens gelegentlich Gespräche mit medizinischem Fachpersonal^f^	0,06 (−0,38; 0,50)	0,790	0,03 (−0,32; 0,38)	0,863	0,12 (−0,15; 0,39)	0,374
Mindestens gelegentlich Nutzung von Informationen der Gesundheitsämter^f^	0,16 (−0,26; 0,58)	0,467	0,12 (−0,29; 0,52)	0,563	−0,20 (−0,48; 0,07)	0,145
Mindestens gelegentlich Nutzung von Fernsehen/Radio aus Ausland^f^	0,57 (0,15; 1,00)	0,008	0,29 (−0,10; 0,68)	0,141	1,22 (0,94; 1,50)	0,000
Mindestens gelegentlich Nutzung von Webseiten von Krankenkassen^f^	0,21 (−0,26; 0,69)	0,379	0,13 (−0,30; 0,57)	0,545	−0,03 (−0,36; 0,30)	0,854
Mindestens gelegentlich Nutzung von Flyern/Broschüren, z. B. vom Arzt^f^	0,05 (−0,47; 0,57)	0,847	−0,01 (−0,49; 0,47)	0,968	−0,22 (−0,59; 0,16)	0,257

*β (95* *%-KI)* Regressionskoeffizient mit 95 %-Konfidenzintervall

^a^Referenzgruppe: weiblich

^b^Referenzgruppe: (Fach‑)Hochschulabschluss

^c^Referenzgruppe: mit Migrationshintergrund

^d^Referenzgruppe: ≥ 150 % Median/Nettoäquivalenzeinkommen

^e^Referenzgruppe: neue Bundesländer, einschl. Ostberlin

^f^Referenzgruppe: keine oder seltene Nutzung der jeweiligen Informationsmöglichkeit

Der Anteil aller *Ungeimpften* nahm, unabhängig von ihrer Impfabsicht, mit steigendem Alter und mit höherem Bildungsgrad ab. Signifikante Geschlechtsunterschiede waren nicht festzustellen. Sowohl bei denjenigen, die beabsichtigten, sich impfen zu lassen, als auch bei denen, die noch unentschlossen waren, ließen sich signifikante Effekte des Migrationshintergrundes und des Haushaltseinkommens feststellen. Der Anteil ungeimpfter Menschen, die impfbereit oder unentschlossen waren, war höher, wenn ein Migrationshintergrund vorlag oder das Einkommen des Haushalts unter 60 % des Nettoäquivalenzeinkommens lag. Dagegen spielten diese Faktoren bei Menschen, die sich nicht impfen lassen wollten, keine Rolle. Sowohl diejenigen, die beabsichtigten, sich impfen zu lassen, als auch Unentschlossene waren in den alten und den neuen Bundesländern etwa gleich stark vertreten. Menschen ohne Impfabsicht sind jedoch in den neuen Bundesländern stärker vertreten.

Unter denjenigen, die sich über TV- und Radiosender aus Deutschland oder in Tageszeitungen und Zeitschriften informiert hatten, fanden sich weniger Menschen, die sich nicht impfen lassen wollten. Dafür war diese Gruppe größer, wenn sie sich in sozialen Netzwerken beziehungsweise über TV- und Radiosender aus anderen Ländern als Deutschland informiert hatte.

## Diskussion

Die Daten der dritten Erhebung der CoSiD-Studie stehen im Einklang mit anderen Studien zur Mediennutzung während der COVID-19-Pandemie, wonach vor allem klassische massenmediale Informationsangebote wie Fernsehen, Radio, Zeitungen, aber auch interpersonale Kontakte und institutionelle Expertise wesentliche Informationsquellen darstellten [21, 37, 48]. Zu berücksichtigen ist allerdings, dass in diesen Studien unterschiedliche Populationen in verschiedenen Phasen der Corona-Pandemie befragt wurden. In der CoSiD-Studie wurden weitere Informationsmöglichkeiten abgefragt, zum Beispiel Webseiten staatlicher Gesundheitseinrichtungen (RKI, BZgA, BMG). Diese wurden am vierthäufigsten zur Information über die COVID-19-Schutzimpfung genutzt, was ein gewisses Maß an Vertrauen in diese Angebote widerspiegelt.

Die Daten der Erhebung stammen aus einem Zeitraum, in dem unter anderem die 3G-Regelung (geimpft, genesen, getestet) im öffentlichen Nah- und Fernverkehr in Kraft trat. Außerdem wurden Auffrischungsimpfungen stark beworben. Dennoch lag der Anteil der Nicht-Geimpften in der CoSiD-Studie noch bei 12,3 % und damit nur unwesentlich höher als im COVID-19-Impfquoten-Monitoring (COVIMO-Studie, ca. 10 %; [12]). Der in der Fachliteratur beschriebene Zusammenhang zwischen soziodemografischen Variablen (insbesondere Alter und Bildungsgrad) und der Inanspruchnahme einer Corona-Schutzimpfung wird durch diesen Beitrag weitgehend bestätigt. Bei Differenzierung nach der Impfabsicht der Nicht-Geimpften zeigte sich, dass insbesondere die Anteile Impfbereiter und noch Unentschlossener höher sind, wenn ein Migrationshintergrund besteht oder niedrigere Einkommensverhältnisse vorliegen. Damit fallen auch diese Ergebnisse ähnlich aus wie in der COVIMO-Studie [12, 23]. Dies verweist darauf, dass es den Menschen in diesen Gruppen bisher weniger gelungen ist, ihre Impfabsicht umzusetzen oder zu einer positiven Impfeinstellung zu kommen. Sie bedürfen daher besonderer Aufmerksamkeit bei der Entwicklung zielgruppenspezifischer Angebote. Bei der Betrachtung der genutzten Informationsquellen zur COVID-19-Schutzimpfung fällt auf, dass sich mehr Menschen ohne Impfabsicht finden, wenn soziale Medien, wie etwa Whatsapp und Instagram, oder TV- und Radiosender aus anderen Ländern als Deutschland genutzt wurden. Das könnte damit zusammenhängen, dass dort häufig Falschinformationen zu finden sind [39–43]. Auch die Rezeption von Informationen zur Impfung in manchen ausländischen Medien hat möglicherweise eine impfkritische Einstellung befördert.

Der vorliegende Artikel zeichnet sich dadurch aus, dass als abhängige Variable nicht nur der Impfstatus untersucht wird, sondern die Gruppe der Ungeimpften hinsichtlich ihrer verschiedenen Impfabsichten differenziert wird. So zeigt sich zum Beispiel, dass es unter Menschen mit Migrationshintergrund mehr Impfbefürworter und Unentschlossene gibt als unter Menschen ohne Migrationshintergrund, während der Anteil der Menschen, die die Impfung ablehnen, sich in beiden Gruppen kaum unterscheidet. Außerdem wurden das Impfverhalten und die Impfabsichten nicht nur in Abhängigkeit von soziodemografischen Merkmalen untersucht, sondern es wurde auch die Nutzung verschiedener Informationsquellen berücksichtigt. Die Analysen zeigen, dass unabhängig von sozialen Unterschieden das Informationsverhalten zusätzliche Erklärungskraft besitzt.

Als Limitationen sind anzuführen, dass die CoSiD-Studie aufgrund ihres Studiendesigns zwar bevölkerungsrepräsentative Aussagen über Impfstatus bzw. -absicht und damit zusammenhängende Merkmale ermöglicht, die Daten allerdings nur querschnittlich erhoben wurden. Dies lässt keine kausalen, sondern lediglich assoziative Schlussfolgerungen zu. Im Regressionsmodell wurden die Einflüsse der sozialen Merkmale und des Informationsverhaltens unabhängig voneinander modelliert und somit keine Interaktions- oder vermittelnden Effekte getestet. Es ist nicht auszuschließen, dass auch die Nutzung verschiedener Quellen von sozialen Merkmalen der Informationssuchenden beeinflusst wird. Die Daten basieren zudem auf subjektiven Einschätzungen und die Ergebnisse können von sozial erwünschtem Antwortverhalten beeinflusst sein.

## Fazit

Die sich nach sozialer Zugehörigkeit unterscheidenden Inanspruchnahmeraten der COVID-19-Impfung weisen auf einen gesundheitskommunikativen Handlungsbedarf hin. Es bedarf zusätzlicher Informationsangebote, die leicht zugänglich und verständlich, zielgruppenspezifisch und dialogorientiert, lebensweltnah sowie seriös bzw. vertrauensbildend konzipiert werden sollten. So werden mehr Menschen in die Lage versetzt, abgesicherte Entscheidungen zu treffen. Auf diese Weise kann am besten mit Mythen und Fehlinformationen aufgeräumt werden, Ängste können gezielt abgebaut und Vorurteile beseitigt werden.

Entsprechend wird auch in der 5. Stellungnahme des ExpertInnenrates der Bundesregierung zu COVID-19 „Zur Notwendigkeit evidenzbasierter Risiko- und Gesundheitskommunikation“ vom 30.01.2022 [49] eine faktenbasierte und handlungsorientierte Information der Bevölkerung in Gesundheitskrisen als zentral eingeschätzt. Dabei sollte der Fokus auf die unentschlossenen Bürgerinnen und Bürger gesetzt, ein breites Spektrum an Informationskanälen genutzt, die Kommunikation aufsuchend gestaltet (z. B. in sozialen Brennpunkten) und Multiplikatorinnen und Multiplikatoren auf regionaler und lokaler Ebene stärker eingebunden werden (siehe Bericht des Sachverständigenausschusses zur „Evaluation der Rechtsgrundlagen und Maßnahmen der Pandemiepolitik“ vom 30.06.2022 [50]).

Zudem ist die Bedeutung der Vermittlung insbesondere digitaler Gesundheitskompetenz zu betonen, damit seriöse Informationen im Internet als solche besser erkannt werden.
